# Emerging Relationships between Exercise, Sensory Nerves, and Neuropathic Pain

**DOI:** 10.3389/fnins.2016.00372

**Published:** 2016-08-23

**Authors:** Michael A. Cooper, Patricia M. Kluding, Douglas E. Wright

**Affiliations:** ^1^Department of Anatomy and Cell Biology, University of Kansas Medical CenterKansas City, KS, USA; ^2^Department of Physical Therapy and Rehabilitation Science, University of Kansas Medical CenterKansas City, KS, USA

**Keywords:** exercise, pain management, neuropathy, inflammation, neurotrophins, dorsal root ganglion

## Abstract

The utilization of physical activity as a therapeutic tool is rapidly growing in the medical community and the role exercise may offer in the alleviation of painful disease states is an emerging research area. The development of neuropathic pain is a complex mechanism, which clinicians and researchers are continually working to better understand. The limited therapies available for alleviation of these pain states are still focused on pain abatement and as opposed to treating underlying mechanisms. The continued research into exercise and pain may address these underlying mechanisms, but the mechanisms which exercise acts through are still poorly understood. The objective of this review is to provide an overview of how the peripheral nervous system responds to exercise, the relationship of inflammation and exercise, and experimental and clinical use of exercise to treat pain. Although pain is associated with many conditions, this review highlights pain associated with diabetes as well as experimental studies on nerve damages-associated pain. Because of the global effects of exercise across multiple organ systems, exercise intervention can address multiple problems across the entire nervous system through a single intervention. This is a double-edged sword however, as the global interactions of exercise also require in depth investigations to include and identify the many changes that can occur after physical activity. A continued investment into research is necessary to advance the adoption of physical activity as a beneficial remedy for neuropathic pain. The following highlights our current understanding of how exercise alters pain, the varied pain models used to explore exercise intervention, and the molecular pathways leading to the physiological and pathological changes following exercise intervention.

## Introduction

Twenty five million Americans are encumbered by acute pain and over 50 million suffer from varying chronic pain syndromes, leading to a medical cost of over $635 billion a year (Gaskin and Richard, 2012). This enormous health plight highlights the need to find novel interventions to reduce the burden of chronic pain. Generally speaking, chronic pain undergoes a progressive movement from peripheral tissues, such as the hands and feet, to the central nervous system which often leads to even more debilitating and chronic effects as the disease progresses (Tesfaye et al., 2013; Jones et al., 2016). The perception of pain is a very broad and complex mechanism to study, having multiple origins including nerve damage, metabolic disease, and numerous others. Each form of pain may be unique not only in its development but also in the treatments necessary to provide relief. Unfortunately, current therapies available for the treatment of these pain states are still associated with pain abatement and do not address underlying mechanisms driving the development of varying forms and levels of sensory discomfort (Schreiber et al., 2015).

Physical activity offers a wide array of benefits and is well documented to help in a myriad of diseases, however the mechanisms by which exercise exerts its benefits are poorly understood. The complexities of understanding how global cross organ communication and changes induce molecular changes to provide benefits in disease makes exercise research often hard to perform on a basic level. However, the clear benefits of exercise provide a strong rational to continue to study this complex intervention.

Nociceptive and neuropathic pain syndromes both receive physiological and behavioral benefits from exercise intervention, even though they are thought to have separate physiological characteristics. Nociceptive pain results from an expected noxious stimulus, while neuropathic pain occurs in the absence of a stimulus, or with a normally innocuous stimulus. The neuronal pathway of nociceptive pain starts with a noxious stimulus detected by a peripheral sensory peripheral terminal, of an Aδ- or C-fiber. The electrical signal is then propagated up through spinal and thalamic pathways to terminate in an appropriate somatotopic region of the cortex (Serpell, 2006). In the case of neuropathic pain, adaptations occur in Schwann cells, satellite cells, the peripheral immune system, spinal microglia, and astrocytes that lead to the development of a painful syndrome when one would not normally exist (Scholz and Woolf, 2007). Important areas to examine in these pain pathways are interneuronal interactions and the molecular and cellular changes that are initiated within them. This is an important aspect of any therapeutic target for pain due to the activity-dependent neuronal plasticity that occurs in the nervous system (Zhuo et al., 2011).

In response to new information about neuronal activity-dependent plasticity, a new and rapidly growing area within both pain research and neural physiology has begun to examine the effects of exercise on peripheral and central nervous system components. However, the scarcity of well-controlled basic research in this area hampers the utilization of exercise as a therapy for neuropathic and other chronic pain syndromes. While exercise intervention is growing quickly as a clinical therapeutic tool for many diseases, its use to reduce pain states is still relatively new and the research available leaves an incomplete picture of the molecular pathways affected. Continued research therefore is vital to gain a better understanding of how exercise benefits the management of various pain syndromes and for the implementation of this therapeutic technique on a broader scale by physicians.

## Sensory pathways sensitive to exercise

A well-established effect of exercise is its activation of afferent sensory nerves from active muscles to the spinal cord. Activity in sensory fibers of working muscles is increased throughout exercise and provides important feedback on the cardiovascular and respiratory systems during physical activity (Mitchell, 1985). One example of afferent nerve activity affected by exercise is the exercise pressor reflex (EPR), which is responsible for the control of blood pressure and heart rate (HR) changes during physical activity through sympathetic nerve activation (O'Leary et al., 1999; Amann et al., 2011). This reflex is partially mediated by the transient receptor potential vanilloid 1 (TRPV1) receptor, the sensory receptor respondent to capsaicin that is stimulated from temperature and pH level changes (Smith et al., 2010). Similar to TRPV1, the acid sensing ion channel 3 (ASIC3) found on sensory nerve terminals in active skeletal muscle is involved in the regulation of arterial pressure through the EPR (Tsuchimochi et al., 2011). However, EPR is additionally modulated by sodium channel (Na_v_) function; these channels in turn, may be modulated through reactive oxygen species levels in the dorsal root ganglion (DRG) (Wang et al., 2011). The EPR sensory pathway crosses over with known painful sensory pathways through the TRPV1, ASIC3 and sodium channel alterations; this cross talk can provide possible avenues by which exercises known benefits may also connect to painful sensory states.

Studies now demonstrate molecular and cellular changes in DRG sensory neurons can be induced by exercise. The benefits of exercise can appear quickly as seen in the improvement in regeneration after nerve injury following as little as 3 or 7 days of exercise (Molteni et al., 2004). This effect may be related to increased production of a number of molecular signals, including brain-derived neurotrophic factor (BDNF), nerve growth factor (NGF), neurotrophin 3 (NT-3), synapsin I (SNAP1), and growth associated protein 43 (GAP43) in sensory ganglia, thereby stimulating axonal growth (Molteni et al., 2004; López-Álvarez et al., 2015). Consistent with this idea, exercised animals display clear alterations in molecular mediators in their DRGs, in large DRG neurons undergo changes in mRNA expression that are associated with neuronal plasticity and apoptosis in response to prolonged exercise, including higher BDNF, NT3, SNAP1, and GAP43 mRNA levels compared to sedentary animals (Keeler et al., 2012). Exercise of animals that have been given a high fat diet suggests that exercise can reverse alterations in neurotrophin changes that are associated with a high fat diet, insulin resistance and pain (Groover et al., 2013). For example, exercise of diabetic mice induces significant increases in GDNF in the spinal cord and sciatic nerve, along with axonal transport in the sciatic nerve (Wright, unpublished observations).

Our own studies suggest that important phenotypic changes can occur in peripheral terminals of epidermal axons in response to exercise (Groover et al., 2013). A high fat diet increases the number of epidermal axons that express tropomyosin receptor kinase A (TrkA), the high affinity receptor for nerve growth factor (NGF). This phenotypic change in peripheral axons corresponds to an increase in pain thresholds of the mice. Importantly, however, continuous exercise reverses this phenotypic change and normalizes pain thresholds (Groover et al., 2013). Finally, Schwann cell proliferation is increased following exercise and may play an important role in the increase in axonal regeneration necessary for appropriate response to peripheral nerve injury The benefits seen with peripheral nerve regeneration are significant enough to achieve improved values in both functional and morphological markers of nerve and motor function post exercise (Bobinski et al., 2011). These studies bolster the idea that axonal regeneration responds positively to exercise.

**Figure 1:**
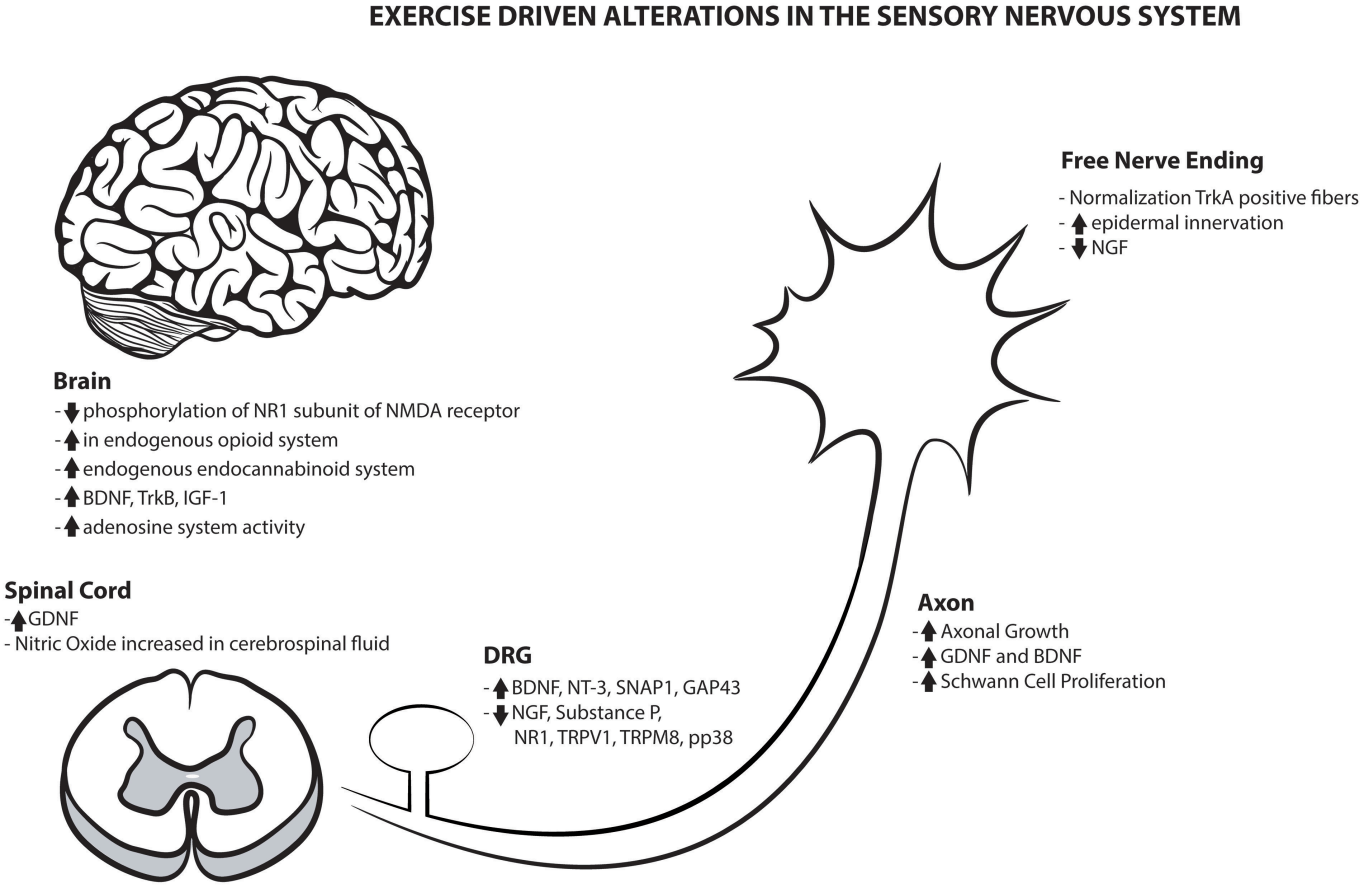
**Exercise driven alterations in the sensory nervous system**. Overview of the numerous positive mechanical alterations that may contribute to the global sensory benefits created with physical activity.

Exercise's benefits are not only limited to the periphery, as they also display a substantial value to the central nervous system. The numerous benefits of exercise on both the peripheral and central sensory nervous system are highlighted in Figure 1. Centrally, the brain imparts bi-directional control of pain processing and pain modulation that alters the transmission and perception of pain and sensation (Denk et al., 2014). The effects of physical activity on this system are grossly understudied and this important central modulation of pain and sensation would benefit by continued examination of the metabolic, inflammatory, and ionic changes within the CNS.

Although only a few studies have been published, regular physical exercise has been reported to prevent the development of chronic muscle pain and exercise induced muscle pain, possibly by reducing phosphorylation of the NR1 subunit of the N-methyl-D-aspartate (NMDA) receptor in the brainstem, modulating nociception and individual experiences (Sluka et al., 2013). Utilizing either aerobic and resistance exercise, an increase in circulating nitrate levels is seen in both the plasma and cerebrospinal fluid, this observation as well as a loss of analgesic benefit of exercise by nitric oxide inhibitors suggest the nitric oxide/cyclic GMP pathway may provide an antinociceptive benefit during physical activity (Galdino et al., 2010a, 2015a,b).

Additionally in the brain, exercise increases the endogenous opioid content in brainstem regions important in pain modulation, suggesting that exercise-induced reversal of neuropathic pain may include an up-regulation of endogenous opioids (Stagg et al., 2011). This may be a key analgesic mechanism as patients with chronic pain display a reduced endogenous pain inhibition system and creating an imbalance between pain modulation systems (Denk et al., 2014). This highlights another benefit of exercise in which it can increase endogenous analgesic systems known to be critically important in modulating pain. However, the endogenous opioid system however has been disputed in its role in modulating internal antinociceptive effects during physical activity (Galdino et al., 2010b, 2014a). This group has instead suggested the endogenous endocannabinoid system is playing a prominent role in the antinociceptive benefits of exercise (Galdino et al., 2014b,c). There is a definitive need to further explore these endogenous systems that are sensitive to exercise and play a prominent role in antinociception.

The primary benefits of physical activity may have an additive effect when paired with pharmacological interventions. One such study reported that the osteoporosis drug risedronate combined with treadmill running had the most efficacious effects on improving bone mineral density and decreasing sensory nerve calcitonin gene-related peptide (CGRP) expression in osteoporotic rats, as compared to rats receiving only the drug (Orita et al., 2010). In conclusion, while exercise is often thought of as a preventative intervention, there also appears to be a clear benefit after injury. The benefits of exercise affect the nervous system at multiple levels and multiple sites associated with sensory function. Emerging evidence is also revealing the molecular pathways that seem sensitive to, including axonal growth, altered neurotrophin levels, and phenotypic changes in both the periphery and central components of the nervous system.

## The relationship of inflammation and exercise

The immune and nervous systems interact substantially in chronic pain states via immune cells, glia and neurons that coordinate immune responses and the excitation of the pain pathway. Many of these interactions include the synthesis and release of inflammatory mediators and neurotransmitters (Ren and Dubner, 2010). When injured, damaged tissue will signal mast cell degranulation and pro-inflammatory cytokine release such as tumor necrosis factor alpha (TNF- α) and interleukin 1 beta (IL1-β). Additional actions include increased NGF signaling via TrkA that enhances substance P release and pain signal propagation in the spinal cord. In response to peripheral tissue injury, immune cells also synthesize and secrete anti-inflammatory cytokines (IL-10 and IL-6), pro-resolution lipid mediators and opioid peptides to suppress the pain from pro-inflammation cytokines (Rittner et al., 2008; Xu et al., 2010). Many of these pro-inflammatory signals are present acutely after exercise, however, chronic examination of these markers post-exercise often show a robust anti-inflammatory signaling cascade in response to these acute pro-inflammatory markers (Woods et al., 2012). However, it is important to note that in non-healthy patients, there is variability in the acute and chronic inflammatory effects. Overall, however, the chronic effects of exercise on inflammation are still viewed as beneficial to reduce inflammatory signaling in disease (Ploeger et al., 2009). For this reason, exercise has been utilized as an intervention that can activate this natural anti-inflammatory mechanism that causes cells to secrete anti-inflammatory cytokines that suppress the pain induced by pro-inflammatory cytokines (Paley and Johnson, 2015).

Numerous cytokines [interleukin 1 alpha (IL1- α), IL1-β, interleukin 2 (IL-2), interleukin 4 (Il-4), interleukin 6 (IL-6), interleukin 10 (IL-10), interferon gamma (IFN-γ), transforming growth factor beta (TGF-β), TNF-α] become active in the spinal cord and DRG during conditions of neuropathic pain (Hopkins and Rothwell, 1995; Ledeboer et al., 2007; Mika et al., 2008; Racz et al., 2008; Wei et al., 2013). Pro-inflammatory cytokines have been implicated in neuropathic and inflammatory nociceptive conditions in a wide array of research (Mika et al., 2013). The most prominently studied inflammation marker, TNF-α, has been implicated as having a key role in both the peripheral and central mechanisms of sensitization to painful stimuli (Leung and Cahill, 2010). Due to their recurring presence during painful stimuli, inflammatory cytokines have been investigated as a sensible target for the explanation of the reduction in allodynia and nociceptive symptoms observed in neuropathic models that utilize endurance and resistance exercise as a rehabilitative technique (Zdziarski et al., 2015).

**Figure 2:**
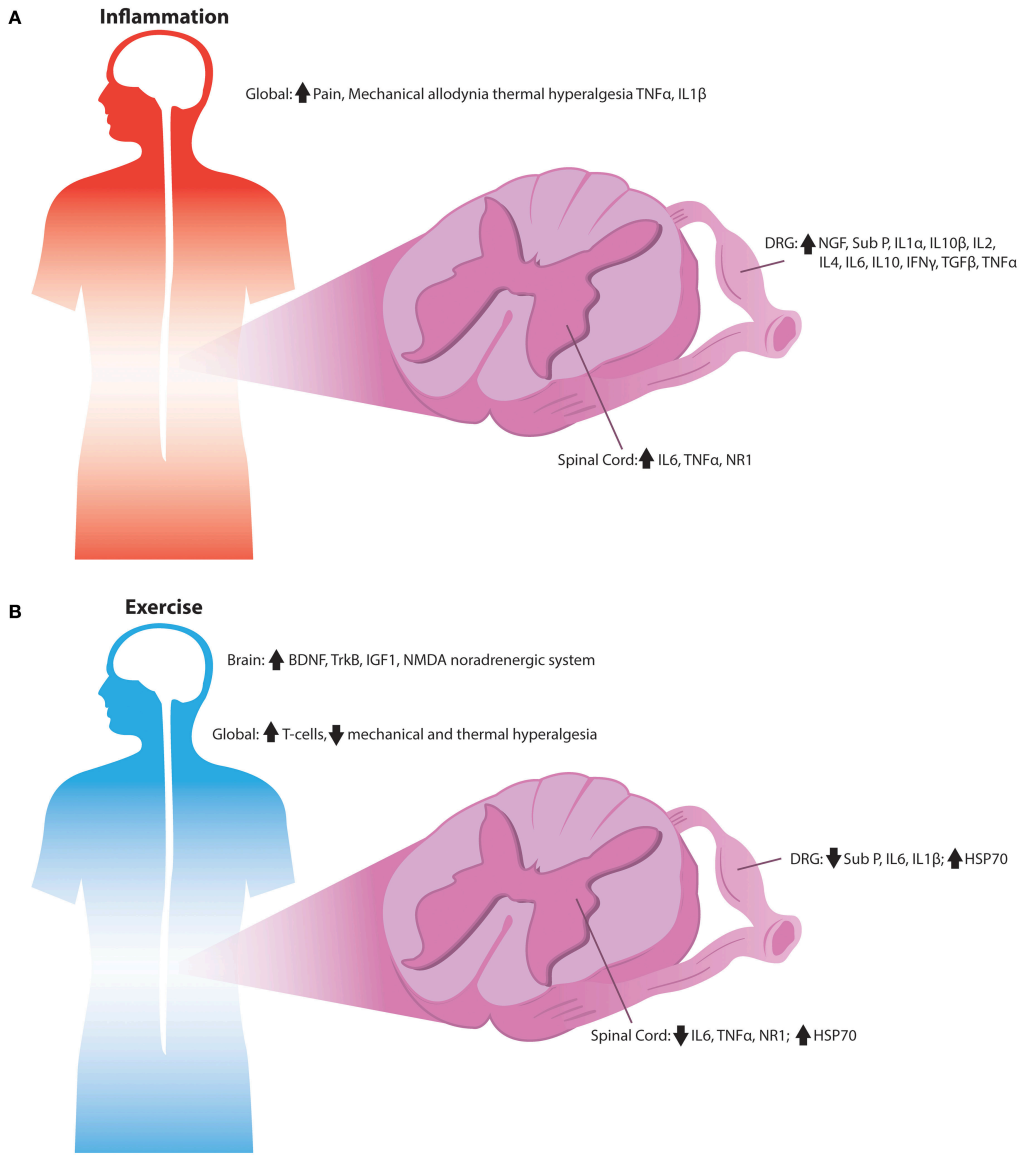
**Signaling alterations associated with inflammation by exercise in the sensory nervous system. (A)** Overview of the inflammatory alterations in the sensory nervous system of rodents. Inflammatory cytokines are increased throughout the DRG, spinal cord, and peripheral tissues during numerous pain states, which is associated with the development of mechanical and thermal hypersensitivity. **(B)** Anti-inflammatory signaling observed in the sensory nerves of exercised rodents. Exercise's anti-inflammatory signals may reduce pro-inflammatory cytokines; while increasing heat shock proteins and T-cells leading to reduced mechanical and thermal hypersensitivity normally associated with inflammation.

A prominent benefit of both endurance and resistance exercise programs is their reduction of pro-inflammatory cytokines and their increase in anti-inflammatory markers as displayed in Figure 2 (Gleeson et al., 2011). The pro-inflammatory acute affects of exercise are proposed to cause a subsequent spike in anti-inflammatory cytokines that are long-lasting after completion of the exercise bout. Regular exercise has been found to decrease inflammatory markers in both young and older humans (Mattusch et al., 2000; Tsukui et al., 2000; Geffken et al., 2001; Colbert et al., 2004). During and after exercise, skeletal muscle increases levels of IL-6, which appears to be responsible for the rise in levels of anti-inflammatory cytokines such as IL-10 and IL-1 receptor agonist (IL-1RA) (Pedersen, 2009). IL-6 has been described as a myokine, a cytokine that is released from muscle fibers during contraction while exerting its effects on other organs (Petersen and Pedersen, 2005). When given as an intravenous infusion, IL-6 provide anti-inflammatory effects similar to a bout of exercise and suppressed pro-inflammatory cytokines such as TNF- α suggesting that IL-6 levels are the cause of anti-inflammatory benefits seen from exercise (Starkie et al., 2003).

Anti-inflammatory markers respond to the rise in IL-6 induced by exercise and have compounding effects that can cause a decrease in allodynia. Increases in IL-10 are able to decrease the expression of pro-inflammatory cytokines and, in-turn, increase the ability of T cells to provide inflammatory responses (Maynard and Weaver, 2008). In mice that exercise via running wheels, T cells were increased in number and associated with a reduction in pro-inflammatory cytokines and an increase in anti-inflammatory cytokines (Wang et al., 2012).

Research showing a decrease in allodynia and hyperalgesia due to exercise intervention suggests that the alterations in painful sensations are strongly influenced by the increase in anti-inflammatory and decrease in pro-inflammatory cytokines. For example, forced treadmill running reduces substance P, IL-6, TNF- α, NR1, and IL-1β levels after the development of mechanical allodynia induced by skin/muscle incision and retraction (Chen et al., 2013b, 2014). Swimming and treadmill exercise decrease mechanical allodynia, cold allodynia, and heat hyperalgesia while also decreasing TNF- α and IL1-β production (Chen et al., 2012; Yoon et al., 2015). Diabetes associated neuropathic pain is markedly reduced by progressive exercise training, possibly mediated by an increase of heat shock protein-72 (HSP72) without increases in TNF- α and IL-6 (Chen et al., 2013a). HSP72 is suggested to have a role in the inhibition of TNF- α and IL-6 as well as many other cytokines (Moseley, 1998). A greater expression of HSP72 and a reduction in diabetes-associated neuropathic pain, including thermal hyperalgesia and mechanical allodynia is seen after exercise (Chen et al., 2013a). HSP72 has an essential role in blocking inflammation and insulin resistance associated with a high-fat diet that can lead to type 2 diabetes (Chung et al., 2008). Extended swimming reduces mechanical allodynia and thermal hyperalgesia in rats with formalin and nerve injury-induced inflammatory pain (Kuphal et al., 2007). In conclusion, the interactions between release of pro-inflammatory cytokines and exercise's anti-inflammatory role via the up-regulation and release of anti-inflammatory myokines provides a mechanism that includes multiple sites and actions by which exercise can benefit overactive pain neurons.

## Experimental studies of neuropathic pain and exercise

Many of these studies discussed above have centered on the ability of exercise to alleviate neuropathic pain associated with diabetes as it is the most prevalent form of neuropathic pain being investigated in hopes of improving diabetic neuropathy (Zilliox and Russell, 2011). Table 1 highlights studies in rodents in which exercise was used as an intervention. A delay in onset of diabetes-associated neuropathy with continuous exercise may be associated to changes in calcium channel function in the DRG allowing for an alteration in nociceptive signaling from the periphery (Shankarappa et al., 2011). Additional benefits such as increased motor nerve conduction velocities occur in diabetic patients, modifying or delaying the natural course of diabetic peripheral neuropathy (DPN) (Balducci et al., 2006). The mechanism by which exercise is alleviating neuropathic pain is still far from known however, and may very well be the combination of a multitude of changes driven by physical activity. This may especially be true in instances where exercise is seen to provide alleviation in differing forms of neuropathy as well as different types of allodynia.

**Table 1:** **Summary of various rodent studies addressing sensory dysfunction associated with pain**.

**Species**	**Mode of exercise**	**Pain model**	**Benefit**	**References**
Rat	Forced running	Skin/muscle incision	↓ Substance P, TNF-α, IL-1β	Chen et al., 2013b, 2014
		Chronic muscle pain	↓ NR1 phosphorylation	Sluka et al., 2013
		Spinal cord injury and acidic saline	↓ Mechanical allodynia	Hutchinson et al., 2004; Sharma et al., 2010
		Sciatic nerve constriction	↓ Heat hyperalgesia and cold allodynia	Chen et al., 2012
		Sciatic nerve crush	↑Schwann cell proliferation	Seo et al., 2009
		Lumbar spinal nerve ligation	↑Endogenous opioids	Stagg et al., 2011
		Sciatic nerve cut	↓ NGF and BDNF	López-Álvarez et al., 2015
		Sciatic nerve cut	Normalized NKCC1 regulation	López-Álvarez et al., 2015
		Osteoporosis	↓ CGRP fibers in bone	Orita et al., 2010
		Paclitaxel-induced neuropathy	↑Epidermal axon innervation	Park et al., 2015
		Streptozotocin	↑HSP72	Chen et al., 2013a
		Streptozotocin	↓ TRPM8, TRPV1, and pp38	Yoon et al., 2015
		Acute antinociception	Activated endogenous cannabinoid system	Galdino et al., 2014b
		Acute antinociception	Activation of nitrous Oxide/cGMP pathway	Galdino et al., 2010a, 2015a
Rat	Swimming	Nerve constriction and inflammation	↓ Mechanical allodynia and heat hyperalgesia	Kuphal et al., 2007; Chen et al., 2012
		Streptozotocin	↓ TNF-alpha and IL-1β	Yoon et al., 2015
		CRPS type I	↑Adenosine	Martins et al., 2013
Rat	Resistance exercise	Acute antinociception	Activated endogenous cannabinoid system	Galdino et al., 2014a
		Acute antinociception	Activated nitrous oxide/cGMP/KATP pathway	Galdino et al., 2015b
Mouse	Running wheel	High fat diet/pre-diabetes	↓ Mechanical allodynia	Groover et al., 2013
		High fat diet/pre-diabetes	↓ TrkA positive fibers	Groover et al., 2013
		High fat diet/pre-diabetes	↓ NGF, ↑BDNF	Groover et al., 2013
		Nerve crush	↑BDNF, NT3, GAP43, and SNAP1 (mRNA)	Molteni et al., 2004
Mouse	Treadmill	Sciatic nerve crush	↑Nerve regeneration	Bobinski et al., 2011

*A range of species, modes of exercises, pain model, and primary outcomes are provided*.

In a type I DPN rodent model, running is able to rescue many different forms of allodynia including mechanical, cold, and heat hyperalgesia. Associated with these forms of allodynia are key molecular markers such as TRPV1 (heat) and transient receptor potential cation channel subfamily M member 8 (TRPM8) (cold), which were also positively altered by forced running (Yoon et al., 2015). Voluntary aerobic exercise has a recovery effect on nociceptive symptoms and behavior developed from early stages of diabetes such as pre-diabetes induced by a high-fat diet (Groover et al., 2013). However, researchers and clinicians must always be careful not to exceed the level of exercise at which activity is no longer a therapeutic tool, as there has been limited evidence that exercise can increase negative outcome variables in some instances, as discussed in the Clinical section of the current review. An over zealous training protocol can lead to the loss of many benefits seen with activity due to the bodies self protection through the downward activation of glial cells in both motor and sensory neurons (Pereira et al., 2015).

A survey of the literature suggests that diabetes is the most researched disease relative to exercise and neuropathic pain; however, physical activity is also a useful therapeutic tool for pain derived from other sources. Paclitaxel-induced neuropathy frequently occurs in patients undergoing chemotherapy and induces a loss of sensation and sensory fiber loss in the skin. Treadmill exercise reduces the symptoms of sensory loss and increases epidermal nerve fiber density in paclitaxel-treated mice (Park et al., 2015). Additional results include the ability of exercise to decrease abnormal levels of detyrosinated tubulin in paclitaxel-treated nerves, highlighting important anti-neurotoxic effects of exercise (Park et al., 2015). Other studies have shown that moderate intensity exercise reduces hyperalgesia and increases in the neurotrophin, neurotrophin 3 (NT-3), which acts in an analgesic fashion in a number of different pain conditions (Sharma et al., 2010). In studies of spinal cord injury associated pain, treadmill training improves sensory function, ameliorated allodynia, and restores normal sensation after within 5 weeks of the spinal cord injury (Hutchinson et al., 2004). Physical activity also decreases the presence of phagocytic and reactive glial cells following spinal cord injury, suggesting that the positive impact of exercise is limited not only to pain scores, but may also lead to improved functional scores and improved neuronal tissue health (Sandrow-Feinberg et al., 2009). This finding suggests that rhythmic, weight-bearing exercise may be an effective intervention to counter spinal cord injury induced allodynia. Finally, studies of pain associated with sciatica report that physical exercise has a negative influence on nociception. Although this model is not a widely utilized model of pain, in this case, exercise producing more hyperalgesia in rats with sciatica than in a control, non-exercised group (Bertolini et al., 2011). Thus, studies demonstrating negative actions of exercise demand that caution be used to address pain with exercise, as all forms of pain do not respond in the same fashion to exercise.

Varying modes and intensities of exercise have been tested to treat neuropathic pain, almost all of which have a positive effect (Hutchinson et al., 2004; Balducci et al., 2006; Kuphal et al., 2007; Sharma et al., 2010; Shankarappa et al., 2011; Stagg et al., 2011; Sluka et al., 2013). However, not all forms and types of exercise provide the same type or degree of benefit, particularly related to the intensity of exercise (Seo et al., 2009). For instance, treadmill running will increase neurite outgrowth with low intensity, but not high intensity exercise levels. However, studies have not rigorously investigated how varying modes of exercise impact a single model and a single sensory dysfunction. This is likely because researchers focus on a single exercise modality throughout their research study for consistency and control among experimental studies.

Regardless of mode, almost all prominent exercise methods demonstrate beneficial effects as seen with the number of studies previously discussed utilizing traditional aerobic and resistance exercise methods, however even less common forms such as swimming provide a benefit to the nervous system. Swimming provides positive results as a therapy for induced nerve injury in rats, reducing both mechanical allodynia and thermal hyperalgesia (Shen et al., 2013). In addition, swimming reduces pain hypersensitivity in a number of experimental models, including formalin and nerve injury-induced animal models of persistent pain, decreasing nerve injury, induced cold allodynia, thermal hyperalgesia in rats, and decreased nerve injury-induced hyperalgesia in mice (Kuphal et al., 2007). The mechanism by which swimming exercise reduces mechanical allodynia may involve endogenous adenosine and adenosine A_1_ receptors (Martins et al., 2013). It is reported that agonists to the adenosine A1 receptor reduce mechanical allodynia in a neuropathic pain model of diabetes, suggesting another possible mechanism in which exercise may reduce pain (Katz et al., 2015). These positive results from swimming offer an extremely attractive exercise modality for patients with neuropathic pain due to the reduced load on pain-affected extremities and problems with coordination many patients, especially in elderly patients. For this reason, additional research in swimming regimens is needed to understand benefits for neuropathic pain, as its utilization could be the best avenue for relief for many patients.

Just as exercise intensity and mode may be key factors in the benefits of physical activity, time of exercise onset and duration may prove to be important as well. Intense short-burst exercise significantly reduced mechanical allodynia in a chronic constriction injury model of neuropathic pain, resulting in better recovery of sensorimotor function (Cobianchi et al., 2010). The relationship of time between the onset of injury and the start of exercise is not clear, however, there are multiple studies that have reported positive results with exercise starting within 1 week of injury. Initiation of treadmill running 3 days after an induced injury had an immediate and long-lasting reduction in pain that was independent of the duration of exercise (Cobianchi et al., 2013). Exercise training beginning 5 days after injury was sufficient to prevent the development of neuropathic pain (Detloff et al., 2014). Also, exercise initiation 7 days after spinal nerve ligation was able to reduce thermal and tactile hypersensitivity (Stagg et al., 2011). These studies suggest that there may be no need to wait for a certain amount of time to pass after injury before the introduction of exercise as a therapeutic aide; however, understanding of how a given injury may affect motor control should be taken into consideration as improper exercise technique can minimize benefits or even increase negative effects of an injury.

## Clinical use of exercise

The use of physical exercise as a therapeutic treatment to specifically address pain is a relatively new and developing field. The majority of research on exercise for peripheral pain syndromes in human subjects is associated with diabetic or pre-diabetic neuropathic pain. The few studies, which have utilized physical activity, are displayed in Table 2. Historically, clinicians may have been reluctant to encourage exercise in patients with diabetic neuropathy due to the risk of possible adverse outcomes such as foot ulcers in insensate feet or increased pain. People with fibromyalgia have expressed exercise as a pain-inducing stimulus, and report an increase in negative symptoms due to exercise, however, cumulatively exercise has been shown to improve patients quality of life (Nijs et al., 2012; Daenen et al., 2015). Additionally, people with painful diabetic neuropathy (PDN) have reported higher ratings of perceived exertion and muscle pain during exercise and no improvements in thermal pain ratings following exercise (Knauf and Koltyn, 2014). Importantly, however, it has been reported by numerous groups that exercise can be performed safely in patients with type 2 diabetic neuropathies and exercise intervention produces a marked improvement in certain nerve functions (Fisher et al., 2007; Kluding et al., 2015).

**Table 2:** **Summary of human studies addressing sensory dysfunction associated with pain**.

**Mode of exercise**	**Pain model**	**Benefit**	**References**
Aerobic exercise	Diabetic neuropathy	↑Motor conduction velocity	Balducci et al., 2006
	DPN and metabolic syndrome	↑innervation	Kluding et al., 2012; Singleton et al., 2015
	DPN	↓ Pain ratings	Kluding et al., 2012
Vibration Platform	DPN	↓ Pain levels and improved gait	Hong et al., 2013
	DPN	↓ Neuropathic pain scale	Kessler and Hong, 2013
	DPN	↓ Pain ratings	Kessler and Hong, 2013

*Modes of exercises, pain model, and primary outcomes are provided*.

Aerobic exercise is an often-studied modality in clinical programs for people with diabetes. Diabetics have experienced a benefit in both motor and sensory neuropathy measures. Aerobic exercise reduces the development of diabetic neuropathy (Balducci et al., 2006), as well as increasing the intraepidermal nerve fiber density (IENFD) and visual analog scale (VAS) pain measure in people with diabetes (Smith et al., 2006; Fisher et al., 2007; Kluding et al., 2012; Singleton et al., 2014, 2015). Similarly, exercise induced improvement in metabolic syndrome patients saw an increase in cutaneous IENFD even though these patients were non-diabetic (Singleton et al., 2015). Physical exercise when paired with diet counseling has resulted in partial cutaneous re-innervation in pre-diabetic individuals, highlighting that exercise may have on early symptoms and possible prevention of neuropathic symptoms (Smith et al., 2006).

With sensory changes, it is important to remember that anatomical changes are not the only factor to examine, functional changes are just as important for clinical implications. Exercise training's benefit through the reinforcement of existent sensorimotor pathways rather than promoting generation of new pathways may be a significant reason to examine functional changes as outcome measures (de Leon et al., 1998). For instance, patients with motor and sensory neuropathy see a gain in strength with exercise training, but only a marginal functional increase (Allet et al., 2010; Song et al., 2011; Mueller et al., 2013; Dixit et al., 2014). These sensorimotor benefits such as improved balance, mobility, and a decrease in peripheral neuropathy, can combine to significant whole measure outcome increases such as quality of life (QOL) (Streckmann et al., 2014). Even with the knowledge that exercise is beneficial in multiple diseases, providing benefits through a multitude of mechanisms, there exists a great limitation in the breadth of knowledge as to how exercise truly exerts its benefit.

One extremely well documented result of exercise on the sensorimotor pathways is the observation of exercise-induced hypoalgesia (EIH) resulting in a myriad of populations and testing conditions (Koltyn and Arbogast, 1998; Koltyn, 2002; Koltyn and Umeda, 2006; Kodesh and Weissman-Fogel, 2014; Koltyn et al., 2014; Vaegter et al., 2014, 2015b). This induction of hypoalgesia occurs independent of exercise mode with benefits occurring with both interval and traditional aerobic exercise, as well as with resistance exercise (Koltyn and Arbogast, 1998; Kodesh and Weissman-Fogel, 2014; Vaegter et al., 2015b). In line with the development of hypoalgesia, numerous groups have reported a reduction in temporal summation of pain (TSP) following physical activity (Koltyn et al., 2013; Naugle and Riley, 2014; Vaegter et al., 2015a). TSP is commonly used to reflect the amount of CNS involved nociception and is often hypothesized as being sensitive to alteration in acute and chronic pain states, suggesting that exercise may be providing a benefit in both the peripheral and central nervous system pathways important for pain.

A unique aspect of clinical trials in human patients is the ability to perform voluntary resistance based exercise as well as aerobic exercise. A study associated with metabolic features of diabetes that combined aerobic and resistance training did not observe an increase in detrimental affects when compared to a program that utilized only one form of exercise (Sigal et al., 2007). This study saw an improvement in glycemic control through measurement of hemoglobin A1C values in elderly adults that completed a moderate intensity weight program. Combining resistance training with balance training and vibration as opposed to aerobic training saw an improvement in balance, muscle strength, and hemoglobin A1C levels when compared to balance and vibration alone (Lee et al., 2013). Resistance training combined with high intensity training significantly improved muscle strength, blood pressure regulation in long-standing, insulin-treated type 2 diabetics with diabetic neuropathy (Praet et al., 2008). Therefore, individuals with type 2 diabetes looking to improve glycemic control through physical activity should be encouraged to perform both aerobic and resistance training. This point needs to be addressed in future clinical studies associated with pain.

Moderate aerobic exercise helps to preserve peripheral nerve function and help to combat health behaviors associated with DPN in type 2 diabetes (Dixit et al., 2014). In adults with and without diabetic neuropathy, aerobic exercise benefited gait changes, reaction times, and balance measures, although it did not reduce the rate of falls in these groups (Morrison et al., 2014). Combination therapy of backward walking and alpha-lipoic acid treatment (ALA) to reduce and prevent free radical damage through antioxidant action was more effective than just ALA alone when examining plantar pressure in patients with DPN, suggesting that just as seen with mammalian models, exercise may have an additive affect when paired with pharmacological agents (Zhang et al., 2014). A 2013 case study showed that a 4-week vibration treatment designed to simulate movement on the feet of patients with diabetic peripheral small fiber neuropathy significantly improved pain levels and gait (Hong et al., 2013). Another study reported that whole body vibration significantly reduced acute pain in the visual analog pain scale and chronic reduction in neuropathic pain scales (Kessler and Hong, 2013). This proposes that movement alone, or simulation of movement, may be able to provide a benefit for painful symptoms. However, there still remains a significant gap in our knowledge about the molecular pathways altered by exercise.

## Conclusions

The use of exercise as a therapeutic tool is a rapidly growing field in biomedical research. However, there is a dire need for increased research into understanding the role of exercise in sensory nerve disorders. The lack of understanding in the pathways affected by exercise and the molecular changes that lead to the benefits seen with exercise is a hindrance to the medical community working to utilize this tool for their patients. It is our expectation that certain types of pain may benefit from exercise, though different mechanisms driving the development of pain can vary. For instance the benefits of exercise on diabetic pain may be influenced by concurrent correction of metabolic abnormalities, while nerve damage associate pain may be associated with local, acute alterations in gene expression and inflammation. Overall however, the prevailing literature suggests that for the vast majority of nerve related disorders; exercise offers a benefit and can be an attractive therapeutic aide for clinicians. However, the clinical use of exercise requires the investment of the patient and their willingness to expend the effort which exercise requires. To motivate patients to exercise, they must perceive there will be approximately two times greater improvement of symptoms than without exercise (Anderson et al., 2015). Clinicians and researchers therefore must continue to examine and highlight the myriad of benefits which exercise provides. Future research should continue to examine the use of exercise in a clinical setting, looking to answer what changes occur in different neural compartments that underlie reductions in pain. Finally, the use of exercise in human subjects in a larger array of diseases will help expose the clinical benefits of exercise for a larger portion of the health care community.

## Author contributions

MC: Primary author, performed literature review which was the basis of the submitted review. PK: Was consulted with over the clinical research included in the review as well as the shaping of the entire article. DW: Corresponding author, primary editor of the review as well as advisor of all work performed by MC.

### Conflict of interest statement

The authors declare that the research was conducted in the absence of any commercial or financial relationships that could be construed as a potential conflict of interest.

## Abbreviations

EPR: exercise pressor reflex
HR: heart rate
TRPV1: transient receptor potential vanilloid 1
ASIC3: acid sensing ion channel 3
DRG: dorsal root ganglion
BDNF: brain-derived neurotrophic factor
NGF: nerve growth factor
NT-3: neurotrophin 3
SNAP1: synapsin I
GAP43: growth associated protein 43
TrkA: tropomyosin receptor kinase A
NGF: nerve growth factor
NMDA: N-methyl-D-aspartate
CNS: central nervous system
CGRP: calcitonin gene-related peptide
TNF- α: tumor necrosis factor alpha
IL1-β: interleukin 1 beta
IL-10: interleukin 10
IL-6: interleukin 6
IL1- α: interleukin 1 alpha
IL-2: interleukin 2
Il-4: interleukin 4
IFN-γ: interferon gamma
TGF-β: transforming growth factor beta
IL-1RA: IL-1 receptor agonist
HSP72: heat shock protein-72
DPN: diabetic peripheral neuropathy
TRPM8: transient receptor potential cation channel subfamily M member 8
NT-3: neurotrophin 3
IENFD: intraepidermal nerve fiber density
VAS: visual analog scale
QOL: quality of life
EIH: exercise-induced hypoalgesia
TSP: temporal summation of pain
ALA: alpha-lipoic acid treatment.
